# Confocal Laser Endomicroscopy in Oncological Surgery

**DOI:** 10.3390/diagnostics11101813

**Published:** 2021-09-30

**Authors:** Patra Charalampaki, Irini Kakaletri

**Affiliations:** 1 Department of Neurosurgery, Cologne Medical Center, University Witten Herdecke, 58455 Witten, Germany; 2 Medical University of Rheinische Friedrich Wilhelms University Bonn, 53113 Bonn, Germany; irinikakaletri@yahoo.de

The therapy of choice in the treatment of abnormalities in the human body, is to attempt a personalized diagnosis and with minimal invasiveness, ideally resulting in total resection (surgery) or turning off (intervention) of the pathology with preservation of normal functional tissue, followed by additional treatments, e.g., chemoradiotherapy or conservative follow-up. A misinterpretation of diagnosis or/and an incomplete surgical/interventional treatment of a pathology, such as a tumor with remaining infiltrative growing cells, increases the risk of recurrence even with adjuvant therapies, decreasing the quality of life and shortening lifetime.

In case of tumor treatment, intraoperative diagnosis and definition of tumor borders are based on the visualization modalities which the interventionist/surgeon uses, as well as on the histopathologic examination of a limited number of biopsy specimens. The interventional and surgical gold-standard visualization tools today are the microscope, endoscope, neuro-navigation, ultrasonography and magnetic resonance imaging. None of these are able to differentiate between tumor and normal tissue on a cellular level but this is exactly what is needed for achieving the highest surgical accuracy in malignant surgical tumor therapy. Therefore, fast biopsies are usually needed for defining and differentiating tumor cells. They are removed when the tumor is exposed but not yet resected. Unfortunately, intraoperative histopathology is often not sufficiently informative. It is either incorrect or leads to frozen artifacts. The biopsies are often non-diagnostic, or the tissue is mechanically destroyed. In addition, sampling errors are possible since the biopsies do not originate from the most aggressive part of the tumor. Finally, the tissue architecture of the tumor can be altered during the specimen preparation. Other disadvantages are the lack of interactivity with the pathologists for careful intraoperative selection of the biopic point of interest and a mean waiting time of approximately 30 min for the result. In summary, optimal surgical therapy is the combination of an accurate diagnosis followed by maximal near total resection and minimal injury of the normal tissue. This could only be achieved if we were able to identify, intraoperatively, cellular structures, and thus differentiate between tumor and normal functional tissue in order to be able to resect the tumor totally and to protect normal tissue. To achieve this goal, we need new, diagnostic, minimally invasive interventional techniques combined and followed by new therapeutic surgical interventional concepts.

The principle of confocal laser endomicroscopy (CLE), based on extreme miniaturization of the microscope imaging head, offers the possibility of in vivo microscopy with subcellular and subnuclear resolution during ongoing intervention. This can be used even for the diagnosis or the surgical treatment as an additional diagnostic modality on a cellular level, with the ability to perform optical biopsies without having the disadvantages described above. A clear visualization of the cytoarchitecture of the cell structure can be achieved with a 400-fold magnification. CLE was introduced in 2004 in gastroenterology as a supplement to the current standard endoscopy for performing optical biopsies [1,2,3,4,5,6,7]. Step by step it has also become a useful diagnostic as well as therapeutic tool in pulmonology [8,9,10], urology [11,12], neurosurgery [13,14,15,16,17,18] and ENT [19,20,21].

CLE has two major advantages when it comes to oncologically and surgically working disciplines. On the one hand, CLE allows intraoperatively the detection and differentiation of a single tumor cell even on a subcellular or subnuclear level (immediate online diagnosis without the need for fast biopsies). On the other hand, the distinction and definition of borders between tumor and normal tissue on a cellular level makes surgical resection much more accurate than ever before. CLE allows surgeons to identify intraoperatively cellular structures in the whole body, and to make a differentiation between cancer and normal cells. The big challenge of CLE is the interpretation of endomicroscopic information, particularly for clinicians who are not confronted with histopathology on a regular basis. Furthermore, the diagnosis can be examiner-dependent, leading to considerable interobserver variability. Therefore, automatic tissue characterization with CLE would support the interventionist/surgeon in establishing diagnosis as well as in guiding robot-assisted intervention procedures. The application and implementation of CLE-assisted intervention in clinical routines would increase not only the diagnostic but also the therapeutic options. In the case of cancer treatment, it could extend the view of the resected borders on a cellular level and, more importantly, automatically protect the functionality of normal tissue on eloquent areas of the human body, due to visibility properties for small nerve structures.

In conclusion, we think that the most innovative approaches for using CLE in surgical oncology disciplines would be as follows:

- In vivo diagnosis and histomorphological representation of various neoplasms in any surgical oncology-working discipline.
- Definition of the boundaries between tumor and normal tissue is possible in every surgical field.
- Possibility of automated tissue detection based on an integrated data algorithm, immediately after visualization of the lesion before any manipulation (on the tissue) has happened.
- The detection of different fluorescent samples, such as 5-ALA, fluorescein, ICG, etc., is possible. Thus, different structures are simultaneously individually stained and differentiated from each other.
- A greater degree of accurate intraoperative resection on a cellular level of any lesion/pathology is now possible, where this opens further prospects for surgical strategies and systematic intraoperative therapy modalities.
- The cellular recognition of microstructures is of immense importance, especially in vital areas such as in the skull base, to the enhanced protection of the cranial nerves and the brain stem against surgical manipulation and resulting potential damage.
- The flexible handle of the CLE is very easy to integrate into the technical equipment of today’s modern operating theatres and could be used, for example, as a microimplemented system in combination with a multimodal, multifunctional endoscope, or be associated with a surgical microscope.
